# Differences in Gut Microbial Diversity are Driven by Drug Use and Drug Cessation by Either Compulsory Detention or Methadone Maintenance Treatment

**DOI:** 10.3390/microorganisms8030411

**Published:** 2020-03-13

**Authors:** Qiaoyan Li, Siqi Chen, Ke Liu, Danfeng Long, Diru Liu, Zhengchao Jing, Xiaodan Huang

**Affiliations:** 1 School of Public Health, Lanzhou University, No. 222 TianshuiNanlu, Lanzhou 730000, China; liqy18@lzu.edu.cn (Q.L.); chensq18@lzu.edu.cn (S.C.); liuk17@lzu.edu.cn (K.L.); longdf@lzu.edu.cn (D.L.); liudiru@lzu.edu.cn (D.L.); 2 Mengzi Center for Disease Prevention and Control, Mengzi 661199, China

**Keywords:** gut microbial diversity, human gut microbiota, compulsory detention participants, MMT patients, drug users, drug cessation, 16S rRNA gene

## Abstract

In this work, we investigate differences in gut microbial diversity driven by drug use or by the widely used methods for drug cessation: methadone maintenance treatment (MMT) and compulsory detention (CD). Methods: 99 participants (28 CD participants, 16 MMT patients, 27 drug users, and 28 healthy controls) were selected using strict inclusion criteria. Nutritional intake and gut microbial diversity were analyzed with bioinformatics tools and SPSS 20.0. Results: Alpha diversity was not significantly different among groups, whereas beta diversity of gut microbiota and nutrient intake were significantly higher among MMT patients. Taxa were unevenly distributed between groups, with drug users having the highest proportion of *Ruminococcus* and MMT patients having the highest abundance of *Bifidobacterium* and *Lactobacillus.* Conclusion: Drug use, cessation method, and diet contribute to shaping human gut communities. High beta diversity among MMT patients is likely driven by methadone use and high nutrient intake, leading to increased orexin A and enrichment for beneficial bacteria, while diversity in CD participants is largely influenced by diet.

## 1. Introduction

Drug abuse and addiction is an urgent social health problem accompanied by several serious diseases and disorders, especially a high prevalence of HIV [1], HCV [2], HBV, and syphilis [3]. Drug abuse has also been shown to lead to harmful behaviors that cause major social problems, such as domestic abuse and drug-related violence [4], depression [5], and adolescent drug abuse [6]. Thus, programs have been developed to promote drug control through law enforcement and abstinence [7] in order to reduce drug use and facilitate the transition to a healthy lifestyle for former drug users. Many options, such as behavioral counselling, medication, medical devices [8], and drug rehabilitation centers [9], have been successfully implemented for treatment of drug addiction. Methadone maintenance treatment (MMT) has been established as a standard method of remediation for heroin dependence [10], and has been reported to decrease heroin use, HIV infection [11], crime [12], and mortality among drug users (DU) [13,14]. In addition, treatment by compulsory detention (CD) in a drug rehabilitation center is also an important modality for drug cessation [9].

Previous studies have evaluated the effectiveness of different drug withdrawal methods by focusing on the alleviation of chronic, severe pain [15], reduction of heroin use and high HIV-risk behaviors [16], and the sleep quality experienced by MMT patients (MP) [17]. Research on CD has been conducted to quantify relapse rates [9], to study the epidemiology of acquired immune deficiency syndrome (AIDS) and evaluate treatment efficacy [18], to examine relationships between drug abuse and mental health [19], and to assess the risk of violence [20]. However, gut microbial diversity has rarely been studied as an indicator of health in evaluating the effects of CD or MMT on rehabilitated or currently recovering drug addicts.

Generally, gut microbes are considered by many researchers to be an additional human “organ” with metabolic functions [21] because of their significant effects on health, such as in host metabolism, nutrition, physiology, and immune function [22]. The structure and function of gut microbial communities have been shown to be influenced by dietary intake [23], diseases [24], drugs [25], probiotics [26], antibiotics [27], exercise [23,28,29], and smoking [30]. For example, cocaine use is associated with impaired nutritional status [31], and opioid use reportedly induces bowel dysfunction [32]. Xu et al. (2017) showed that *Prevotella*, *Ruminococcus*, and *Roseburia* are found in higher abundance in patients with substance use disorders compared to healthy controls (HC) [33]. Characterization of gut microbiota in rats treated with methamphetamine showed that Ruminococcaceae and Bacillaceae were more abundant in the rats with methamphetamine-induced conditioned place preference compared with the control group [34]. Therefore, gut microbial diversity may be shaped by addiction-associated behaviors and these communities can potentially function as biological indicators to evaluate the health of people with a history of drug use, such as current, former, and recovering drug users.

The Honghe Prefecture of Yunnan Province is a region notorious in China for the prevalence of drug use. Here, in our current study, we selected this location as a survey site to study gut microbial diversity in DU, CD, and MMT study subjects. Given the differences in lifestyle among current and former drug users, the physiological effects of narcotics and psychotropics, and previous findings showing the influence of drugs on gut microbiota, we hypothesized that in addition to the influence of drugs, the means of cessation may also provide a strong contribution to shaping gut microbiomes. To explore the differences between current and former drug users, we used questionnaires to characterize dietary structure and high-throughput sequencing to characterize the microbial communities of individual participants. We then performed correlation analysis to identify relationships between diet, drug use, and microbial community diversity and composition, especially focusing on differences between patients undergoing methadone treatment, CD participants who underwent more than one-year compulsory drug detention treatment, and current drug users. Data generated in this study can facilitate the improvement of methadone maintenance treatment, compulsory detention treatment, and other therapies for former drug users that promote drug rehabilitation compatible with maintenance of gut microbial community diversity and stability, as well as the alleviation of gut-associated disorders in the DU, CD, and MP groups.

## 2. Materials and Methods

### 2.1. Study Subjects and Screening Conditions

Ninety-nine participants from the Honghe Prefecture, Yunnan Province, comprised the full cohort, including 28 CD participants treated in a compulsory isolation drug rehabilitation center for one or two years, 16 MMT patients (MP) using methadone chronically as an alternative therapy for illicit drug addiction, 27 current DU participants with narcotic (heroin) and psychotropic (methamphetamine) drug use disorders, and 28 HC participants working in the Mengzi City Center for Disease Prevention and Control, who were enlisted as control subjects. All selected participants conformed to the following screening standards [35]: (I) no history of acute illness; (II) no history of major chronic diseases (cancer, malignant tumours, kidney disease, heart disease, diabetes, or liver disease); (III) no incidents of acute gastrointestinal disease (constipation, diarrhoea, or inflammatory bowel disease) in the past three months and no history of chronic gastrointestinal diseases (abdominal tuberculosis, colorectal cancer, or gut malignant tumours); (IV) no family history of major acute or chronic diseases; (V) no antibiotic, antiviral, antifungal, analgesic, or anti-inflammatory drugs were taken in the past three months; (VI) no other chronic diseases (autoimmune and allergic diseases); (VII) free from any infectious diseases (AIDS, hepatitis b, hepatitis c, tuberculosis, and syphilis). Additionally, MMT was maintained for a minimum of one month in MP. The MP group only obtained 16 qualified candidates due to the stringency of the screening conditions. After screening, fecal samples were collected from the subjects.

### 2.2. Dietary Nutrition Intake Survey

A dietary frequency questionnaire was designed [36] to investigate the dietary habits of the participants in Honghe Prefecture of Yunnan Province. Each participant’s dietary intake within a month prior to collecting feces was investigated, and 12 typical foods (grains, vegetables, fruits, poultry, livestock meat, seafood, milk, beans, eggs, nuts, condiments, sweets) [37] consumed by participants were analyzed. It is important to note that the diet is uniform for CD participants who are treated in a compulsory isolation drug rehabilitation center. CDGSS 3.0 (developed by Peking Union Medical College & West China Center of Medical Sciences, Beijing, China) nutritional analysis software (which relies on the China Food Composition Database by Chinese Center for Disease Prevention and Control) was used to calculate the total energy and nutrients (i.e., proteins, carbohydrates, fats, and dietary fibers) as proportions of the daily intake of 12 typical food reported in surveys. Principal component analysis (PCA) of the dietary structures among the four groups was carried out to examine the influences of types and amounts of dietary intake.

### 2.3. Fecal Sample Collection and DNA Extraction

Fresh excrement was transferred by the subjects into sterile collection bottles. Immediately upon receipt, the fecal samples were placed on ice and stored at −80 °C until further analysis. The feces (250 mg per sample) were weighed using a high-precision electronic balance with windproof glass. Genomic DNA was extracted from the feces using a QIAamp DNA stool mini kit (QIAGEN, Hilden, Germany). A Nanodrop 2000 (Thermo Fisher Scientific, Waltham, MA, USA) was applied to test the quantity and quality of the extracted DNA. The integrity and concentration of the DNA samples was observed via 1% agarose gel electrophoresis.

### 2.4. Polymerase Chain Reaction (PCR) and High-Throughput Sequencing

PCR was conducted using a TopTaq DNA polymerase kit (Transgen, China) to amplify the V3-V4 region from the bacterial 16S rDNA gene with the primer sets Primer F (Illumina adapter sequence 1 + CCTACGGGNGGCWGCAG) and Primer R (Illumina adapter sequence 2 + GACTACHVGGGTATCTAATCC). Three 1-μL aliquots from each DNA sample were used as a template for amplification according to the manufacturer’s protocols. Illumina index sequences were specifically designed to identify each DNA sample using the following barcoded primers: Fwd 5′-AATGATACGGCGACCACCGAGATCTACACXXXXXXXXACACTCTTTCCCTACACGACGCTCTTCCGATCTCTG-3′ and Rev 5′-CAAGCAGAAGACGGCATACGAGATXXXXXXXXGTGACTGGAGTTCAGACGTGTGCTCTTCCGATCTGAC-3′, in which X indicates a unique barcode for each sample. The amplified products were checked by agarose gel electrophoresis and purified with magnetic beads. All PCR products were sequenced by MiSeq reagent kit version 3 (Illumina, San Diego, CA, USA). Similarly, detailed sequencing methods can be found in the following literature [25,38,39,40,41,42].

### 2.5. Gut Microbiome Analysis and Statistical Analysis

In order to maximize the quantity and quality of reliable sequences, the following protocols were performed. TrimGalore (version 0.4.2) was used to remove bases with a terminal mass of less than 20, any adapter that might be included, and short sequences with length less than 100 bp. After merging via FLASH2 software [43] and removal of low-quality sequences, the primer sequences were trimmed using Mothur (version 1.41.1) [44]. The final clean reads were obtained by deleting the remaining sequences that were <100 bp in length or had an error rate of total bases more than 2 via USEARCH (version 10.0). High-quantity and high-reliability sequences were aggregated into operational taxonomic units (OTUs) with 97% similarity. The raw reads were deposited into the European Nucleotide Archive (accession number PRJEB36803) [http://www.ebi.ac.uk/ena/data/view/PRJEB36803]. Mothur (version 1.41.1) was used for taxonomic assignment of OTUs at the species level with a credibility of >80% annotation based on the Ribosomal Database Project (RDP) classifier [45]. Analysis of variance (ANOVA) was used to determine statistically significant differences in microbial community diversity among the four groups. R software (version 3.4.3) was used to compute the diversity indices of Chao1, observed species, abundance-based coverage estimator (ACE), Shannon and Simpson indices, and coverage in the four groups. The Kruskal–Wallis rank sum test was used to compare differences among the four groups. Rarefaction curves were used to evaluate the sequencing depth.

Principal coordinate analysis (PCoA) based on the Jaccard distance was used to examine differences in gut microbial community structures for all samples through the relative abundance of OTUs. Permutational multivariate analysis of variance (PERMANOVA) were used to determine the significance of intergroup differences. The biomarkers of the four groups were defined by linear discriminant analysis (LDA) effect size (LEfSe) with LDA score larger than 2 and *p*-value less than 0.05 as the standard for significance. Venn diagrams were used to screen out the OTUs that were specific to each group or common between groups. In order to perform reliable appraisals of unique bacterial comparison among different groups, OTUs with relative abundances greater than 0.01% were screened for ANOVA, LEfSe, and Venn diagrams [46,47]. The correlation between gut microbes and dietary factors was evaluated by Pearson correlation analysis with an absolute correlation coefficient greater than 0.3 as the screening threshold. SPSS 20.0 was used to analyze statistical differences with a *p*-value < 0.05 as cut-off for significance.

### 2.6. Ethic Approval and Consent to Participates

All procedures performed were approved by the Medical Ethics Committee of the School of Public Health in Lanzhou University (20180910-1). Participants received detailed information about the study and informed consent was obtained.

## 3. Results

In order to determine differences in human gut microbial community structure related to the process of recovery from substance abuse, we analyzed the demographic data, drug abuse behavior data, diet data, and fecal microbiome data from four experimental groups, including former drug users who ceased use at least one year previously through compulsory detention treatment (CD), current drug users (DU), patients in recovery undergoing methadone maintenance treatment (MP), and healthy control subjects (HC). Ninety-nine participants, including 28 CD, 16 MP, 27 DU, and 28 HC, were enrolled in this study following the guidelines for inclusion.

### 3.1. General Demographic Characteristics among All Groups and Drug Abuse Behaviour in CD, MP, and DU Participants

General demographic information of the four groups is shown in Table S1. The four groups showed significant differences in gender, age, and educational level (*p* ≤ 0.001–0.001; chi-square test). Metadata regarding drug abuse behaviour among CD, MP, and DU study subjects showed significant differences among these three groups, notably in the duration of drug abuse (in months), drug type, and frequency of forced cessation (*p* ≤ 0.001–0.004; chi-square test) (Table S2).

### 3.2. Dietary Intake Data for Each Group

#### 3.2.1. Higher Nutrient and Energy Intake Reported in MP Group

We first examined the dietary intake of individual subjects, then compared these diets to determine if they were different between groups. The average daily intake of 12 typical foods among the four groups is presented in Table 1. This analysis revealed that with the exception of poultry, the consumption of these foods was significantly different among the groups. The consumption of seafood in CD was higher than that in DU (*p* = 0.005), while consumption of sweets in CD was lower than that in DU (*p* = 0.030). The intake of milk, livestock meat, and nuts was higher in MP and HC than in CD and DU (*p* ≤ 0.001–0.025), but consumption of eggs was lower in MP and HC than in CD and DU (*p* ≤ 0.001–0.002). Vegetable intake was lower in HC than in CD and DU (*p* < 0.001). Higher consumption of fruits was observed in MP than in CD and DU (*p* = 0.001–0.003). The MP group showed higher intake of grains, beans, and vegetables compared with HC (*p* = 0.001–0.008). The daily intake of grains was higher in MP and DU than in HC (*p* = 0.004–0.008).

The available energy and the dietary proportion of energy derived from each macronutrient (proteins, fats, and carbohydrates) among the four groups are shown in Table 2. The total energy (i.e., the energy from all three macronutrients) and the proportion of fat-derived energy were significantly different (*p* ≤ 0.001–0.036) among the four groups, although no significant differences in the proportions of energy derived from proteins and carbohydrates (*p* = 0.079–0.088) were observed among groups. Total energy and protein- and carbohydrate-based energy in the MP group were the highest compared with other groups (*p* ≤ 0.001–0.046), and in HC these values were higher than that in CD (*p* ≤ 0.001–0.016). The energy derived from fat was higher in MP than in CD and DU (*p* ≤ 0.001–0.008); however, the CD group received a higher percentage of energy from fat than did the MP group (*p* = 0.025). Analysis of the differences in dietary fiber consumption among the four groups revealed that the MP group had the highest intake compared with other groups (*p* ≤ 0.001–0.033).

#### 3.2.2. PCA of Dietary Structure Indicates Broad Separation of MP Group Compared to Other Groups

**Figure 1:**
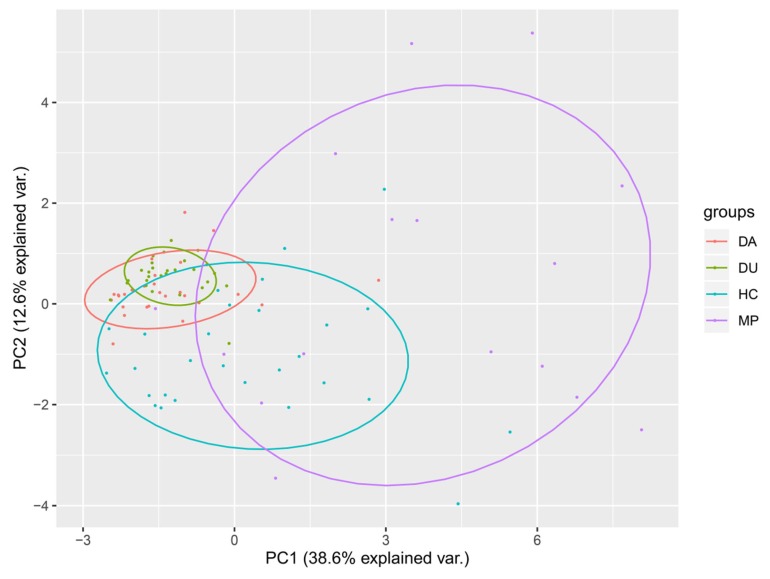
Principal component analysis (PCA) of dietary structure among DU, CD, MP, and HC groups. Note: CD = compulsory detention participants; MP = MMT patients; DU = drug users; HC = healthy controls.

Principal component analysis of the dietary intakes (including food types and quantities) for the four groups was conducted in order to identify similarities or differences between the subject groups that potentially contribute to diversity and microbiome structure (Figure 1). The diets of MP subjects clustered separately from those of DU, whereas the diets of CD subjects overlapped almost completely with that of DU. Only HC appeared to share substantial commonalities with all other groups.

### 3.3. Fecal Microbiome Data of Four Groups

#### 3.3.1. Alpha Diversity was not Significantly Different among Groups

A total of 9,914,426 raw reads were obtained from 99 fecal samples. After removal of adapters and low-quality sequences, 8,584,636 (86.59%) clean reads remained, with a median length of 417 bp and a size range of 100–453 bp. Using 97 percent similarity as a cut-off for OTU binning, a total of 1056 OTUs were clustered within the 8,584,636 total reads. All rarefaction curves reached a plateau, indicating that sequencing depth was sufficient (Figure S1).

After determining the presence, relative abundance, and commonality of microbiota within and among different subject groups, we then examined the alpha diversity of these communities between groups to better understand if drug use or different recovery treatments significantly affected the stability or imbalance of gut microbial communities. Alpha diversity indices were used to evaluate the species richness and diversity of the fecal microbial community, and the Kruskal–Wallis rank sum test was used to determine significance. Differences in the richness and abundance of OTUs were non-significant between groups in comparisons of observed species, Chao1, ACE, and Shannon and Simpson indices, although differences in the coverage index among the four groups were significant (*p* = 0.035) (Figure S2).

#### 3.3.2. Distribution of Taxa Is Significantly Different across Groups in Beta Diversity

**Figure 2:**
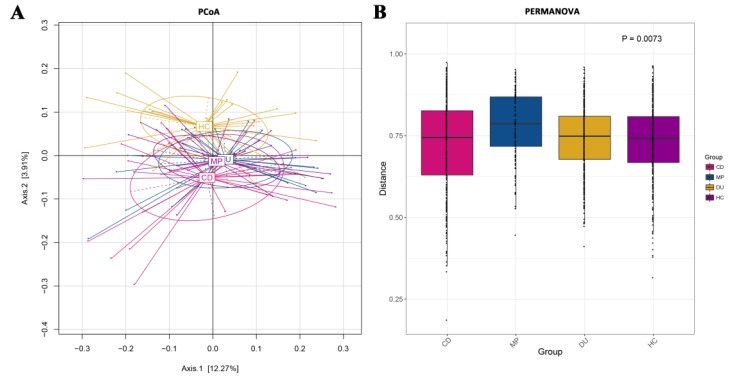
Analysis of beta diversity for bacterial communities among DU, CD, MP, and HC. (**A**) Principal coordinate analysis (PCoA) based on the Jaccard distances. (**B**) Permutational multivariate analysis of variance (PERMANOVA) using distance matrices. CD = compulsory detention participants; MP = MMT patients; DU = drug users; HC = healthy controls.

To better understand how drug use and recovery may affect microbial diversity within individuals, we examined beta diversity across samples. Using principal coordinate analysis (PCoA) based on the Jaccard distance (Figure 2A), we found that bacterial community structure differed among the four groups in some critical respects, such that HC and CD separated along axis 2, with some overlap. However, microbiota of DU and MP were highly similar, and both overlapped with and were intermediate between CD and HC along axis 2. PERMANOVA analysis of the distance matrices (Figure 2B) showed that these differences among the four groups were significant (*p* = 0.0073).

Following identification of the taxa present in different samples, we then examined which OTUs with relative abundances greater than 0.01% are unique to or shared between gut communities of drug users, recovering users, former users, and non-users to better understand the relationship between microbiota and the effects of different treatments for drug use. A total of 281, 280, 281, and 277 OTUs were identified in the CD, MP, DU, and HC groups, respectively (Figure S3). Venn diagrams of unique and common OTUs for each group indicated that the four groups shared 251 OTUs, representing 84.80% of the 296 detected OTUs. Among the remaining 45 OTUs, 6 OTUs were found only in one group, including 1 endemic OTUs each in the CD and DU groups, 4 OTUs unique to HC, and no unique OTU in MP. The remaining 39 OTUs were shared between two or three subject groups.

Analysis with LEfSe further revealed significant differences in the distribution of taxa with relative abundances greater than 0.01% across the four groups (Figure S4). At the phylum level, Cyanobacteria chloroplast and Actinobacteria were identified in highest abundance in the MP group (LDA = 3.224–3.378, *p* < 0.001). At the genus level, *Lactobacillus*, *Streptococcus*, *Veillonella*, *Bifidobacterium*, *Intestinibacter*, and *Fusicatenibacter* were all highly abundant in MP (LDA = 2.824–3.583, *p* ≤ 0.001–0.005). *Aestuariispira* had a high abundance in HC (LDA = 3.108, *p* = 0.012). However, *Ruminococcus*, *Roseburia*, *Collinsella*, and *Succinivibrio* were enriched in DU (LDA = 3.043–4.366, *p* ≤ 0.001–0.018), while *Alloprevotella*, *Erysipelotrichaceae incertae sedis*, and *Flavonifractor* were found in high abundance in CD (LDA = 2.778–3.905, *p* = 0.004–0.047) (Figure S4B).

#### 3.3.3. Significant Enrichment for Specific Genera in MP Compared to CD, DU, and HC Groups

**Figure 3:**
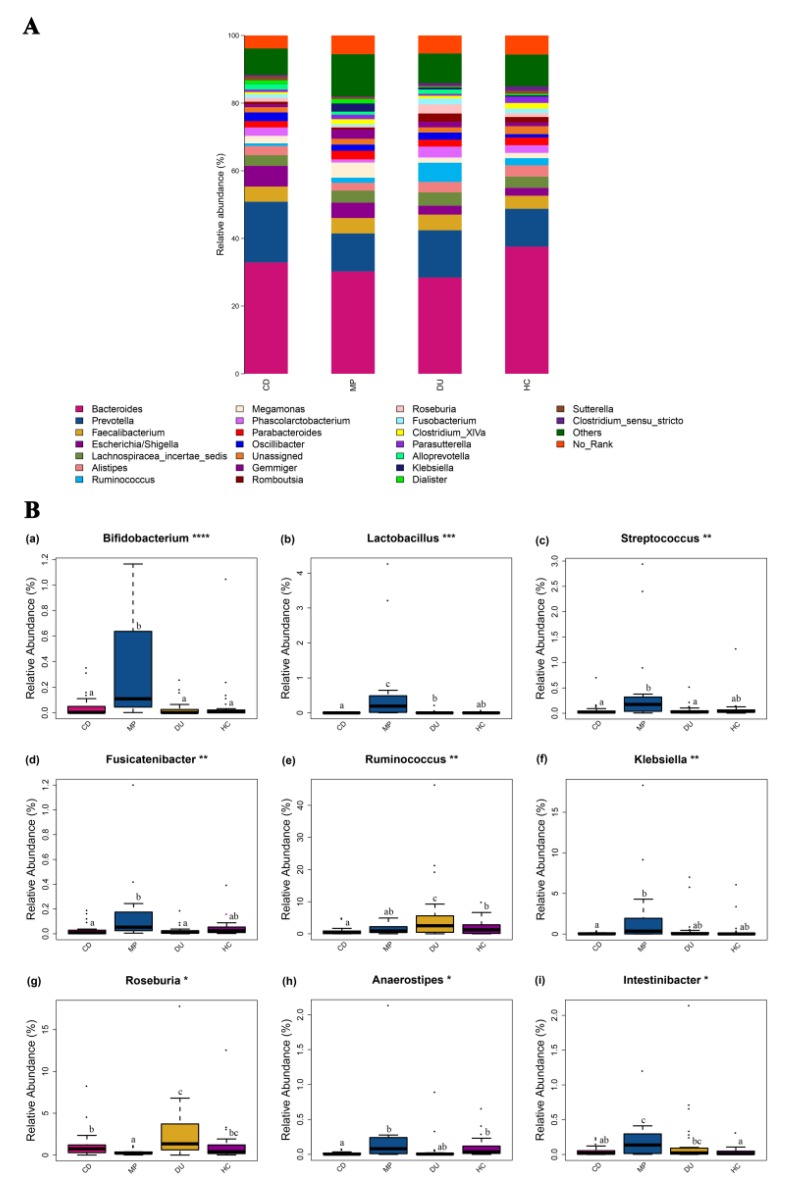
Analysis of community composition and analysis of variance (ANOVA) comparing differences in the abundance of OTUs at the genus level among DU, CD, MP, and HC. (**A**) Relative abundance of bacterial genera among different groups. (**B**) Box plot comparison of taxa with the most significant differences. Note: * indicates statistical significance identified by ANOVA analysis: **** *p* < 0.0001; *** *p* < 0.001; ** *p* < 0.01; * *p* < 0.05; ^a–c^ superscripts indicate significant differences between mean values by pairwise comparison between groups, *p* < 0.05. CD = compulsory detention participants; MP = MMT patients; DU = drug users; HC = healthy controls.

In order to better understand differences in gut microbiota potentially associated with drug use and recovery, we first analyzed the composition of the communities in individuals from each group to identify specific taxa that were unique to each population, as well as the prevalence of these taxa within microbial communities. A total of 1056 OTUs were detected among all samples, spanning 17 phyla, 25 classes, 35 orders, 70 families, and 163 genera. The four most abundant species were from the Bacteroidetes (55.97%), Firmicutes (33.88%), Proteobacteria (8.02%), and Fusobacteria (1.25%) phyla, representing 99.12% of all detected OTUs (Figure S5A). The most commonly observed genera across samples were *Bacteroides* (32.59%), *Prevotella* (13.86%), and *Fecalibacterium* (4.36%), which comprised 50.81% of all classifiable OTUs. Similar patterns of distribution for the predominant genera were observed across the four subject groups (Figure 3A), although one-way ANOVA and *q*-test for pairwise comparison revealed significant differences between the groups in species abundance at the phylum and genus levels (Figure S5B, Figure 3B). Two phyla in particular were significantly different among the four groups. The gut microbiota in DU were significantly enriched for Firmicutes compared with that in CD (*p* = 0.004). Furthermore, MP showed a higher proportion of Actinobacteria compared with CD and HC (*p* = 0.005–0.014) (Figure S5B).

Additionally, twelve genera were found in significantly different abundances among the four groups, including *Bifidobacterium*, *Lactobacillus*, *Streptococcus*, *Fusicatenibacter*, *Ruminococcus*, *Klebsiella*, *Megasphaera*, *Roseburia*, *Haemophilus*, *Anaerostipes*, *Intestinibacter*, and *Sporobacter* (significantly different genera by pairwise comparison are included in Figure 3B and significantly different genera identified only by ANOVA analysis are included in Figure S6). *Roseburia* was more abundant in DU than in CD (*p* = 0.034), but was least abundant in MP (*p* = 0.001–0.037). Among the four subject groups, DU also showed the highest proportional abundance of *Ruminococcus* (*p* = 0.001–0.038) and this genus was significantly lower in CD than in HC (*p* = 0.013) (Figure 3B).

In general, however, MP contained the highest relative abundance of several taxa, and in particular, *Bifidobacterium* and *Lactobacillus* were found in the highest abundance among the four groups (*p* = 0.001–0.020). Similarly, *Streptococcus* and *Fusicatenibacter* were found in higher levels in MP than in CD and DU (*p* = 0.021–0.043). The abundance of *Klebsiella* from MP was significantly higher compared to that in CD (*p* = 0.007) (Figure 3B).

In some cases, taxa had significantly higher representation in multiple groups. For example, *Anaerostipes* were present in higher proportions in MP and HC compared to CD (*p* = 0.001–0.005), while *Intestinibacter* were more highly abundant in MP than in CD and HC (*p* = 0.008–0.010). These findings indicate that MP group had a greater abundance of most significantly different taxa compared to other subject groups (Figure 3B).

#### 3.3.4. Specific Genera Are Significantly Correlated with Dietary Intake

**Figure 4:**
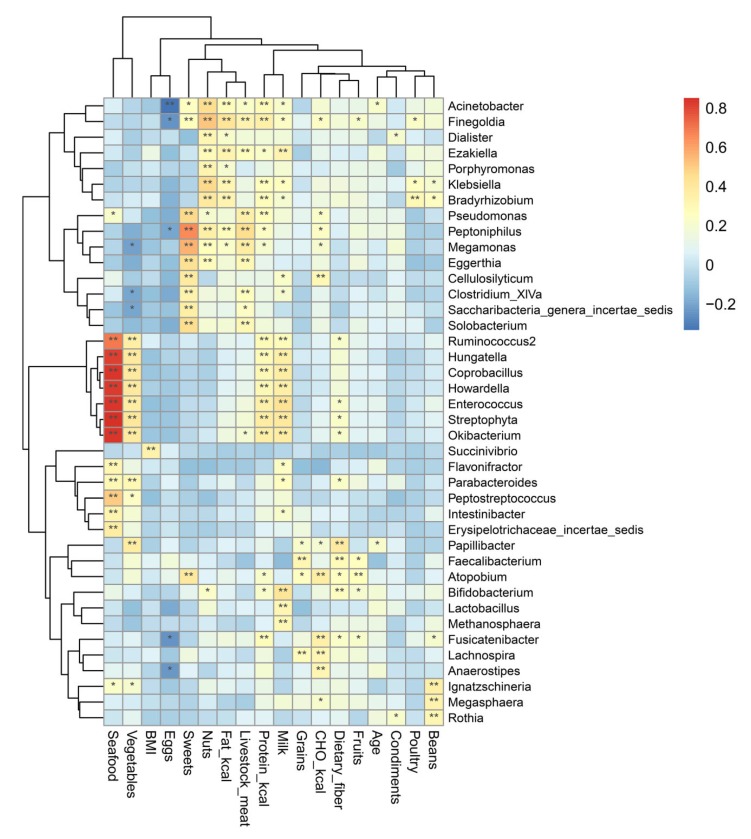
Correlation heatmap between gut microbiota at the genus level and dietary intake. Note: * *p* < 0.05, ** *p* < 0.01; *p*-value was calculated by using Pearson correlation.

To identify a potential relationship between dietary nutrient intake and gut microbiome composition and diversity, a heatmap was constructed to identify any correlations between dietary intake and gut microbiota at the genus level, using a correlation coefficient >0.3 or <−0.3 (Figure 4). In addition, BMI and age were also analyzed for correlations with microbiome composition. We found a positive association between BMI and an abundance of *Succinivibrio* (r = 0.340, *p* < 0.001). High intake of protein-based energy had a positive relationship with *Streptophyta*, *Okibacterium*, *Enterococcus*, and *Finegoldia* (r = 0.348–0.366, *p* < 0.001) compared to energy derived from fat, which was positively associated with *Klebsiella*, *Ezakiella*, *Finegoldia*, and *Bradyrhizobium* (r = 0.317–0.420, *p* < 0.002). In contrast, energy from carbohydrates was positively correlated with the presence of *Lachnospira*, *Anaerostipes*, *Fusicatenibacter*, *Cellulosilyticum*, and *Atopobium* genera (r = 0.306–0.373, *p* < 0.003).

Specific foods also showed correlations with some genera. For example, the abundance of *Papillibacter* was positively associated with the consumption of dietary fiber (r = 0.380, *p* < 0.001), grain intake had a positive association with *Fecalibacterium* (r = 0.313, *p* < 0.002), while milk intake was positively correlated with *Lactobacillus*, *Bifidobacterium*, *Coprobacillus*, *Streptophyta*, *Hungatella*, *Okibacterium*, *Howardella*, *Enterococcus*, *Ezakiella*, and *Methanosphaera* (r = 0.301–0.436, *p* < 0.003). There was a negative association between egg intake and the abundance of *Acinetobacter* (r = −0.333, *p* < 0.001). The consumption of livestock meat was positively associated with *Megamonas*, *Pseudomonas*, *Solobacterium*, *Eggerthia*, *Finegoldia*, and *Peptoniphilus* (r = 0.300–0.454, *p* < 0.003). Poultry intake had a positive association with *Bradyrhizobium* (r = 0.302, *p* < 0.003). The abundance of *Parabacteroides*, *Ruminococcus2*, *Flavonifractor*, *Intestinibacter*, *Coprobacillus*, *Streptophyta*, *Hungatella*, *Erysipelotrichaceae incertae sedis*, *Okibacterium*, *Howardella*, *Enterococcus*, and *Peptostreptococcus* were all positively associated with the consumption of seafood (r = 0.306–0.851, *p* < 0.003).

Additionally, *Megasphaera*, *Rothia*, and *Ignatzschineria* were correlated with the intake of beans (r = 0.308–0.354, *p* < 0.002). Vegetable intake was positively correlated with the presence of *Ruminococcus2*, *Coprobacillus*, *Streptophyta*, *Hungatella*, *Papillibacter*, *Okibacterium*, *Howardella*, and *Enterococcus* (r = 0.361–0.409, *p* < 0.001). Nut intake was positively related with the abundance of *Klebsiella*, *Dialister*, *Porphyromonas*, *Acinetobacter*, *Finegoldia*, *Bradyrhizobium*, and *Peptoniphilus* (r = 0.310–0.512, *p* < 0.002). The abundance of *Megamonas*, *Clostridium XlVa*, *Saccharibacteria genera incertae sedis*, *Cellulosilyticum*, *Atopobium*, *Pseudomonas*, *Solobacterium*, *Eggerthia*, and *Peptoniphilus* all exhibited a positive association with the intake of sweets (r = 0.336–0.655, *p* < 0.001).

## 4. Discussion

In this study, we compared the diversity of gut microorganisms among four groups consisting of former drug users treated by compulsory detention (CD), current drug users (DU), recovering drug users undergoing methadone maintenance treatment (MP), and control subjects (HC) with no history of drug use to determine whether different drug cessation treatments contribute to shaping the composition or structural dynamics of these communities. Through our initial drug abuse behavior survey for participants, we focused on the use of narcotics, psychotropics, and combined use of both narcotic and psychotropic drugs. Responses to the survey indicated that the MP group, comprised of older participants on average, had the highest rate of narcotics abuse, initially due to heroin addiction, but subsequently due to methadone treatment for heroin dependence [10]. Strict inclusion criteria also led to a high representation of participants who used nasal drug delivery, or “snorting”, due to the increased risk of HIV infection through drug injection [48,49].

Dietary intake has been considered one of the largest contributing factors governing shifts in the composition and prevalence of gut microbiota. The reliability of correlations between diet and structure of microbiota depend on self-reporting, which may be inconsistent among individuals and especially among those who may have been conditioned through self-harming habits (i.e., drug use) to supply inadequate information. However, we found no anomalies in the data that would suggest wild inaccuracies related to their self-reporting of diet. Education level may also contribute to the personal habits of the participants in ways that were not evident, simply through close examination of diet and microbiome structures in these individuals who were identified by their drug use. Future studies may examine how education level may be correlated with diet or behavioral patterns, such as smoking or regular exercise, which potentially select for specific gut microbes [23,28,29,30].

We found that the consumption of sweets was lowest in the CD group due to the uniform diet and stringent health standards of the drug rehabilitation center. The total energy from the three macronutrients was the highest among subjects in the MP group, specifically including the consumption of milk, livestock meat, beans, fruits, and nuts compared to consumption of these foods by the CD and DU subjects. These results were consistent with several previous studies. For example, one study has shown that serum levels of orexin A in male heroin addicts receiving MMT are substantially higher than in healthy subjects [50], and that intracerebroventricular administration of orexin A can enhance appetite [51]. In contrast, lower median orexin levels were observed in opiate addicts during opiate withdrawal compared with healthy subjects [52]. Thus, high levels of orexin A among MP patients can potentially enhance appetite and increase food intake, leading to the higher average nutrient intake compared to other groups.

Previous studies examining the gut microbial diversity in humans and animal models of substance addiction have shown ambiguous or contradictory data for alpha diversity. For example, Volpe et al. (2014) found no significant differences in alpha diversity in comparisons of the fecal microbiomes of cocaine users and cocaine non-users [53]. Other work showed that drug addicts have higher Chao1 indices and more observed species compared with healthy subjects [33]. However, Wang et al. (2018) revealed that morphine treatment decreases alpha diversity in mice [38]. Except for the coverage index, our present study showed no significant differences in alpha diversity among these four groups using measures of Chao1, ACE, observed species, and Shannon and Simpson indices, in agreement with Volpe et al. (2014) [53].

ANOVA and LEfSe analysis revealed that OTUs belonging to Actinobacteria, *Klebsiella*, and *Streptococcus* were significantly enriched in the MP subjects compared to other groups. A relatively recent report on fecal microbiome composition among patients with major depression showed a high abundance of Actinobacteria [39], *Klebsiella*, and *Streptococcus* [54]; we speculate this similarity may be related to the prevailing mental disorders that afflict MP subjects, as reported in previous studies of methadone maintenance patients [55,56], although further study would be required to explore this possibility. However, it should be noted that the identification of *Klebsiella* and *Citrobacter* might be confused, depending on which library is used as a reference database [47].

Bacteroidetes and Firmicutes dominated the human gut microbes. In this study, Bacteroidetes (2,180,793 reads) and Firmicutes (1,320,089 reads) comprised 89.85% of all sequences, although the DU subjects had a significantly higher proportion of Firmicutes than the CD group. This finding is comparable to that of Scorza et al. (2019), in which they found that cocaine and phenacetin treatment groups had higher Firmicutes compared with healthy people [25]. We also found that *Bifidobacterium* and *Lactobacillus* had a higher relative abundance in the MP group than in the CD and DU groups, likely related to a selection for probiotic lactic acid bacteria induced by a higher consumption of milk and dairy in the MP group than in the other two groups. Predictably, we observed that milk intake was positively correlated with *Bifidobacterium* and *Lactobacillus*. In light of the many studies showing that probiotics perform many beneficial, physiological functions, increased milk and dairy intake may contribute to maintaining gut health by enriching for these taxa [57,58,59]. Interestingly, we found a very low but significant correlation between condiments (including fermented products, such as soy sauce) and stimulants (such as green tea) with some taxa. Given that these contributions were significant but the effect on overall microbiota structure is ambiguous, future work will more closely scrutinize the relationship between some of these foods and their regular consumption on the microbiota of individuals.

*Ruminococcus* has been established as an essential genus for degradation of recalcitrant starch and cellulose [60,61]. In this study, the DU group had the highest abundance of Ruminococcaceae OTUs compared with the other groups. Xu et al. (2017) reported that *Ruminococcus* OTUs were more abundant in DU compared to HC [33]. Ning et al. (2017) also found that Ruminococcaceae were more abundant in rats with methamphetamine-induced conditioned place preference than in the control group [34]. Furthermore, high abundance of Ruminococcaceae was reported to have a positive relationship with anxiety but was inversely correlated with place memory [62], which negatively impacts cognitive function [34]. Thus, the high abundance of *Ruminococcus* in the gut may be connected to impaired memory and cognitive function for DU subjects, although any relationship between *Ruminococcus* and cognitive function would require closer scrutiny using isolated strains in a model system.

In contrast to alpha diversity, analysis of β-diversity showed significant differences among the four groups, with the MP-associated microbiota clustering together with DU, and both of those groups separated from CD and HC samples (Figure 2A). These differences in local diversity could be potentially driven by current drug use, which distinguishes the DU and MP groups from the CD and HC groups. However, PCA of the dietary structures among the four groups showed a clustered distribution of DU and CD, with the MP diet grouped apart from other groups (Figure 1). Thus, a more likely explanation for the differences in β-diversity may be that direct drug influence (and opioids in particular [63]) in conjunction with the structured diet of MP subjects, which differs in both type and quantity from other groups (Table 1), together enrich for divergent bacterial communities between the drug-using and non-drug-using study participants. Among drug users, however, diet may have less influence on microbiome diversity, since PCA analysis showed substantial overlap in dietary structure between DU and other groups, suggesting that drug usage has the strongest effects on microbial community diversity. The wide separation among individuals of the MP group in the dietary structure PCA may be related to the extremely wide range of food types and overall much higher quantities consumed by this group, a potential side effect of methadone induced-stimulation of orexin A, which is reported to increase appetite [51]. Furthermore, the large differences in the average consumption between MP and other groups contribute to this effect. However, the inter-individual differences in microbiome were not commensurately wide, and closer scrutiny of the individual dietary components may shed light on how specific components affect the distribution of microbes.

Pearson analysis was conducted to explore the significance of the effects of diet on gut microbial community composition among the four groups. Thus, we determined that carbohydrate-derived energy was positively associated with the presence of *Lachnospira*, *Anaerostipes*, and *Fusicatenibacter.* These genera, all of which belong to Lachnospiraceae, may potentially provide inhibitory function against colon cancer in humans through butyric acid production [64]. By extension, the consumption of some carbohydrates in appropriate quantities may enrich for these taxa, and thereby may contribute to protecting against colon cancer in humans [64]. Carbohydrate-based energy and the abundance of *Fusicatenibacter* were higher in the MP group than in the DU group, implying that MMT may play a role in enriching for beneficial gut bacteria that produce butyric acid and potentially act against colon cancer [65,66]. In the current study, the consumption of dietary fiber was positively associated with the abundance of *Papillibacter* (belonging to Class Clostridia), which has been reported to be associated with loss of total fat or body mass, independent of the dietary group [67].

## 5. Conclusions

Although previous studies on gut microbiota have explored the effects of drug use on microbial diversity and community composition, we have focused on identifying changes in microbiota specifically driven by either of the two most widely used methods for drug cessation: compulsory detention treatment and methadone maintenance treatment [68,69,70]. Significant differences in β-diversity and the predominant taxa among current, former, and never-use drug users indicate that drug addiction and subsequent withdrawal might lead to shifts in human gut microbiota. The gut microbiota of current drug users appear to be most strongly influenced by drug usage, characterized by high abundance of Ruminococcaceae OTUs, which has been previously correlated with increased risk of impaired memory and cognitive function. Patients undergoing methadone maintenance treatment exhibited significantly altered gut microbiota compared to current and former drug users, possibly due to the influence of both direct administration of the opiate methadone and dietary changes that led to increased orexin A and enrichment for bacteria such as *Bifidobacterium*, *Lactobacillus*, and *Fusicatenibacter.* These changes suggest that the MMT cessation method may induce inadvertently beneficial effects on gut health, whereas the gut microbiomes of CD participants who underwent compulsory detention treatment, as with healthy control subjects, appear to be more strongly influenced by diet. In addition, the abundance of Ruminococcaceae OTUs were decreased among the MP and CD treatment groups, suggesting that either treatment may potentially decrease the risk of impaired memory and cognitive function for current drug users. This work provides valuable data on the composition of microbiota in drug addicts compared to former drug users and those currently in cessation, providing a foundation for improved understanding of the commonalities and differences that can be exploited in the development of treatments for the effects of withdrawal, especially those related to digestive disorders.

## Figures and Tables

**Table 1:** Average daily intake of 12 typical foods among DU, CD, MP, and HC (Median (Q1, Q3)).

Food	CD Intake (g)	MP Intake (g)	DU Intake (g)	HC Intake (g)	*p*-Value
Grains	528.50 (521.00, 721.00)^ab^	707.50 (498.25, 937.50)^b^	629.00 (571.00, 741.00)^b^	479.50 (277.50, 686.00)^a^	0.013 *
Milk	0.00 (0.00, 0.00)^a^	77.00 (7.50, 461.25)^b^	0.00 (0.00, 8.00)^a^	13.00 (0.80, 167.25)^b^	<0.001 *
Eggs	35.00 (35.00, 35.00)^b^	15.00 (7.00, 33.50)^a^	35.00 (35.00, 35.00)^b^	14.50 (7.30, 30.00)^a^	<0.001 *
Livestock Meat	50.00 (50.00, 62.00)^a^	127.00 (57.00, 166.50)^b^	50.00 (50.00, 52.00)^a^	90.00 (55.50, 126.00)^b^	<0.001 *
Poultry	8.00 (0.00, 21.00)	11.00 (0.00, 37.30)	5.00 (0.00, 20.00)	13.00 (6.50, 60.00)	0.056
Seafood	6.00 (2.00, 20.75)^b^	10.05 (1.25, 28.00)^b^	2.00 (0.00, 3.00)^a^	7.50 (3.00, 19.50)^b^	0.002 *
Beans	30.00 (30.00, 30.00)^a^	102.00 (24.75, 232.25)^b^	30.00 (30.00, 30.00)^a^	24.00 (11.00, 87.25)^a^	0.012 *
Vegetables	283.00 (283.00, 283.00)^b^	364.00 (160.50, 565.00)^b^	283.00 (283.00, 283.00)^b^	186.50 (91.75, 276.75)^a^	<0.001 *
Fruits	70.00 (40.00, 127.50)^a^	215.00 (95.25, 371.75)^b^	70.00 (5.00, 137.00)^a^	134.00 (42.00, 183.75)^ab^	0.003 *
Nuts	0.00 (0.00, 1.00)^a^	14.50 (3.50, 31.50)^b^	0.00 (0.00, 3.00)^a^	3.50 (0.00, 11.75)^b^	<0.001 *
Condiments	0.00 (0.00, 22.75)^a^	87.50 (0.00, 991.25)^b^	0.00 (0.00, 250.00)^ab^	25.00 (0.00, 179.00)^b^	0.046 *
Sweets	0.00 (0.00, 5.25)^a^	4.00 (0.00, 179.25)^b^	10.00 (0.00, 30.00)^b^	8.50 (0.00, 43.50)^b^	0.044 *

Note: The condiments category contained tea, juice, soy sauce, and monosodium glutamate; the sweets included cake, bread, biscuits, and candy; * indicates statistical significance among groups; ^a–b^ superscripts indicate significant differences between mean values by pairwise comparison between groups, *p* < 0.05; *p*-values were calculated using Kruskal–Wallis rank sum test. CD = compulsory detention participants; MP = MMT patients; DU = drug users; HC = healthy controls.

**Table 2:** Energy derived from three macronutrients and dietary fiber among DU, CD, MP and HC (Median (Q1, Q3)).

Nutrients	CD	MP	DU	HC	*p*-Value
Total energy (kcal)	1074.00 (928.25, 1358.25)^a^	3203.00 (1752.50, 3735.25)^c^	1230.00 (1055.00, 1374.00)^a^^b^	1732.50 (1215.25, 2217.75)^b^	<0.001 *
Protein (kcal)	158.00 (130.50, 189.75)^a^	414.50 (284.50, 494.50)^c^	160.00 (149.00, 180.00)^a^^b^	235.50 (161.50, 297.00)^b^	<0.001 *
Fat (kcal)	273.50 (243.75, 365.00)^a^	533.00 (373.75, 887.75)^b^	297.00 (257.00, 396.00)^a^	416.00 (262.50, 555.75)^ab^	<0.001 *
Carbohydrates (kcal)	607.00 (544.00, 771.50)^a^	1849.00 (1178.75, 2688.00)^c^	753.00 (646.00, 850.00)^a^^b^	941.50 (747.25, 1334.75)^b^	<0.001 *
Protein (% energy)	14.00 (14.00, 15.00)	13.00 (11.00, 15.75)	13.00 (12.00, 14.00)	13.00 (12.00, 15.75)	0.088
Fat (% energy)	27.00 (25.00, 28.00)^b^	20.50 (14.50, 25.00)^a^	26.00 (23.00, 28.00)^ab^	23.50 (19.25, 32.00)^ab^	0.036 *
Carbohydrates (% energy)	59.00 (57.25, 60.75)	66.50 (55.75, 74.75)	61.00 (59.00, 64.00)	61.00 (53.25, 67.00)	0.079
Dietary fiber	6.00 (5.00, 8.50)^a^	17.00 (6.25, 25.50)^b^	6.00 (5.25, 6.75)^a^	7.00 (5.00, 11.75)^a^	<0.001 *

Note: * indicates statistical significance among groups; *p*-value was calculated using Kruskal–Wallis rank sum test; ^a–c^ superscripts indicate significant differences between mean values by pairwise comparison between groups, *p* < 0.05. CD = compulsory detention participants; MP = MMT patients; DU = drug users; HC = healthy controls.

## Supplementary Materials

The following are available online at https://www.mdpi.com/2076-2607/8/3/411/s1. Figure S1. Rarefaction curves of each sample. Figure S2. Alpha diversity indices for DU, CD, MP, and HC. Figure S3. Venn diagram of unique and shared OTUs among the DU, CD, MP, and HC groups. Figure S4. Linear discriminant analysis (LDA) effect size (LEfSe) analysis of signature taxa for DU, CD, MP, and HC. Figure S5. Analysis of community composition and ANOVA comparing differences in the abundance of OTUs at the phylum level among DU, CD, MP, and HC. Figure S6. ANOVA comparison of differences in OTUs at the genus level among DU, CD, MP, and HC. Table S1. General demographic information for DU, CD, MP, and HC subjects (n (%)). Table S2. Analysis of drug abuse behavior among CD, MP, and DU.

## Abbreviations

MMT	methadone maintenance treatment.
PCR	polymerase chain reaction.
OTUs	operational taxonomic units.
RDP	ribosomal database project.
ANOVA	analysis of variance.
ACE	abundance-based coverage estimator.
PCoA	principal coordinate analysis.
PCA	principal component analysis.
PERMANOVA	permutational multivariate analysis of variance.
LDA	linear discriminant analysis.
LEfSe	linear discriminant analysis effect size.
BMI	body mass index.
CD	compulsory detention (participants).
MP	MMT patients.
DU	drug users.
HC	healthy controls.
