# Supplementary material for: Differences in Gut Microbial Diversity are Driven by Drug Use and Drug Cessation by Either Compulsory Detention or Methadone Maintenance Treatment
Source: Microorganisms. 2020 Mar 13;8(3):411. doi: 10.3390/microorganisms8030411 (PMC7143234; doi:10.3390/microorganisms8030411)
Supplement: Supplementary file 1 [file microorganisms-08-00411-s001.pdf]

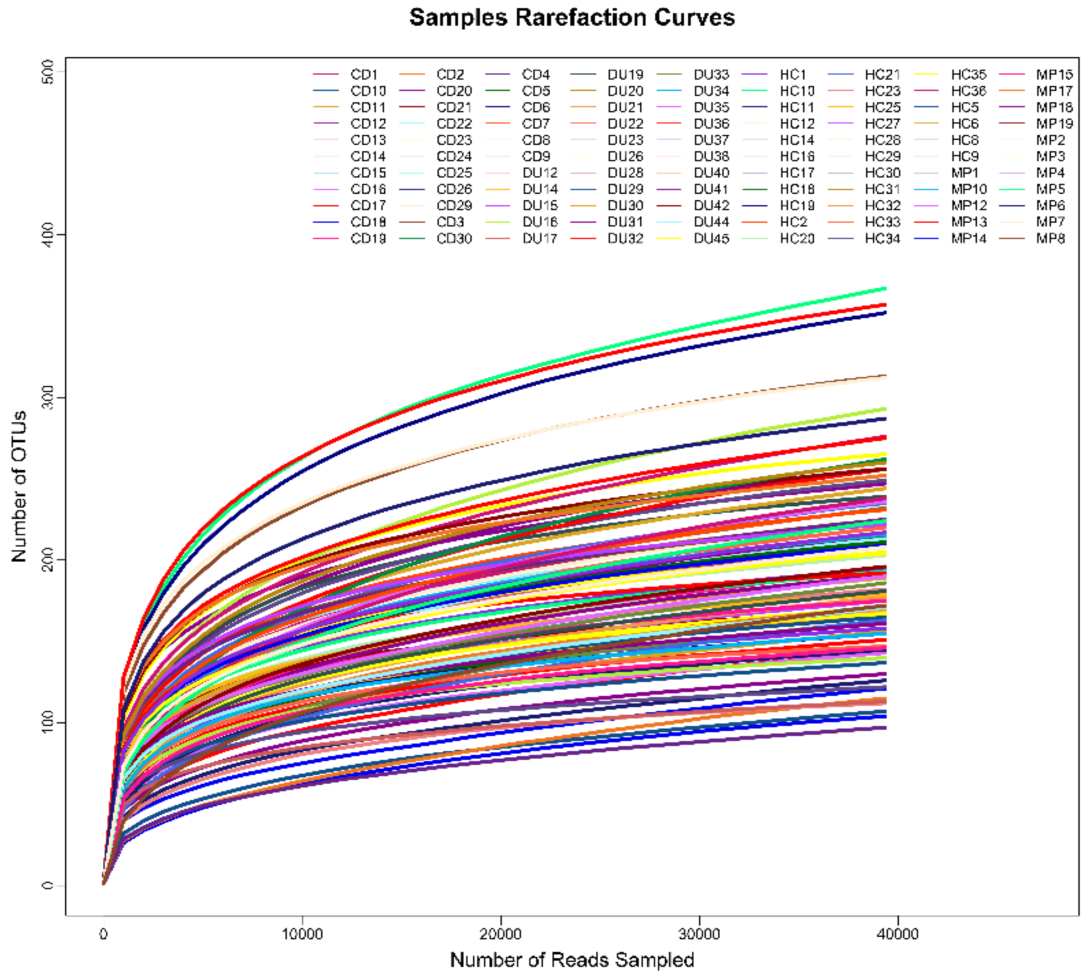

Figure S1. Rarefaction curves of each sample. (CD = compulsory detention participants; MP = MMT patients; DU = drug users; HC = healthy controls).

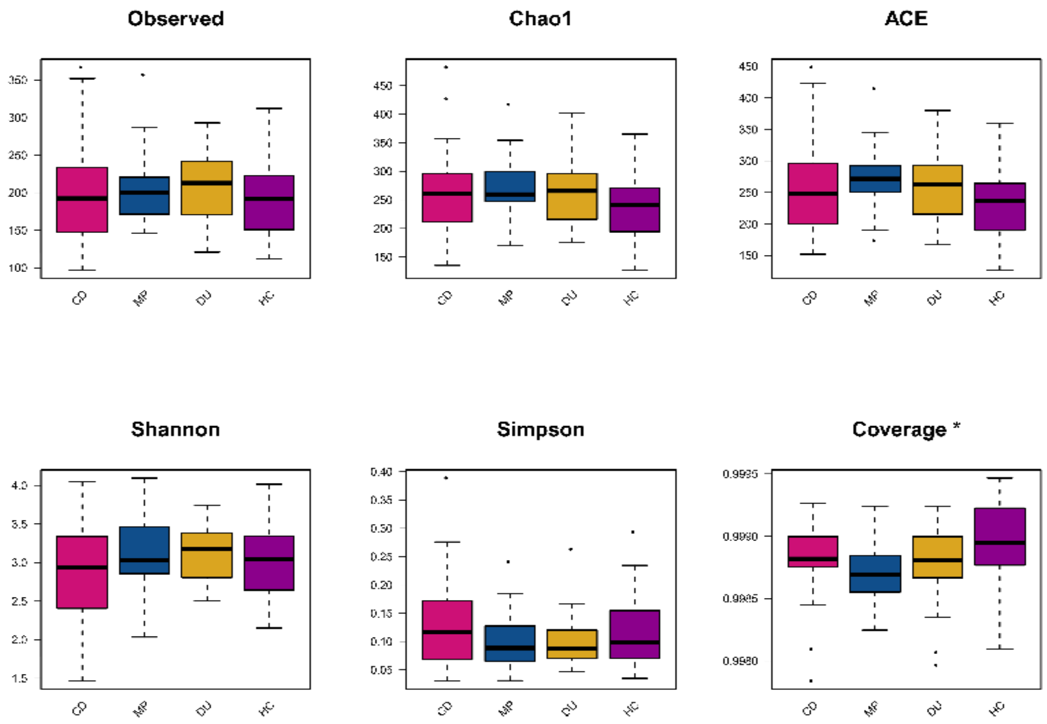

Figure S2. Alpha diversity indices for DU, CD, MP, and HC. (CD = compulsory detention participants; MP = MMT patients; DU = drug users; HC = healthy controls; ACE = abundance-based coverage estimator).

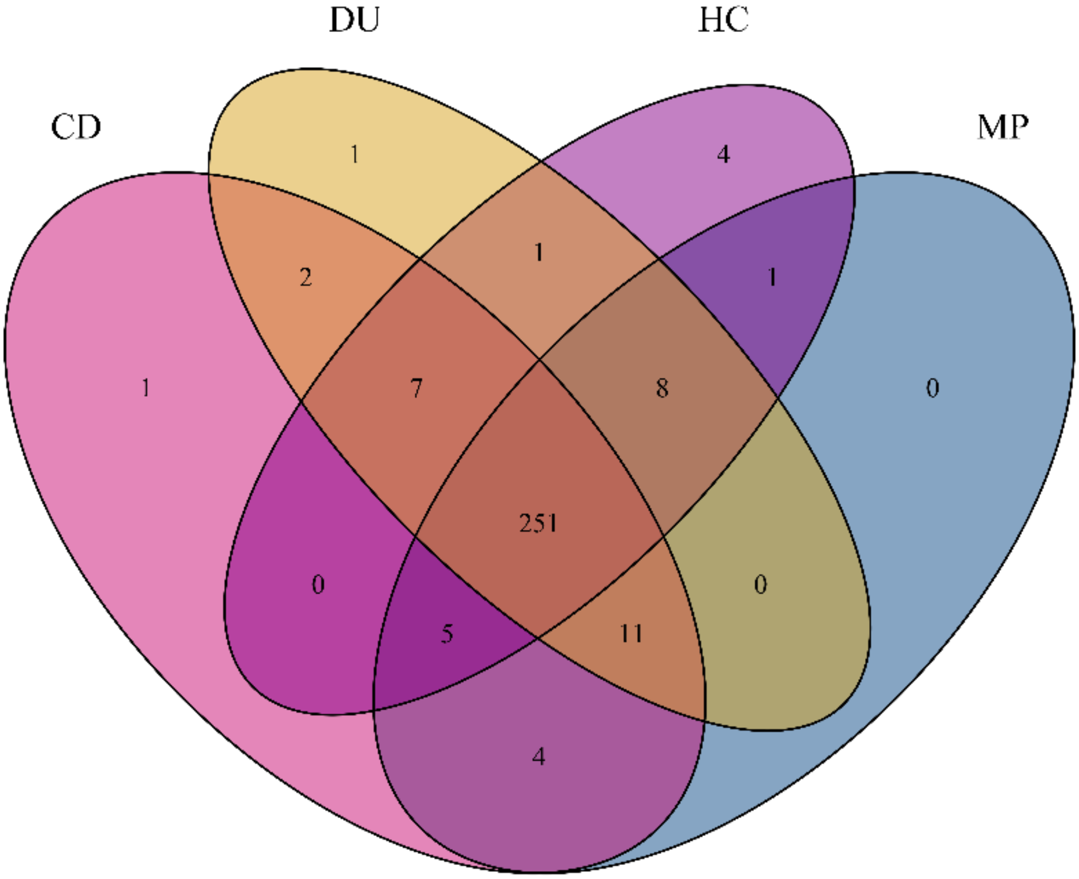

Figure S3. Venn diagram of unique and shared OTUs among the DU, CD, MP, and HC groups. The overlapping areas represent common OTUs. Non-overlapping areas represent unique OTUs. (CD = compulsory detention participants; MP = MMT patients; DU = drug users; HC = healthy controls).

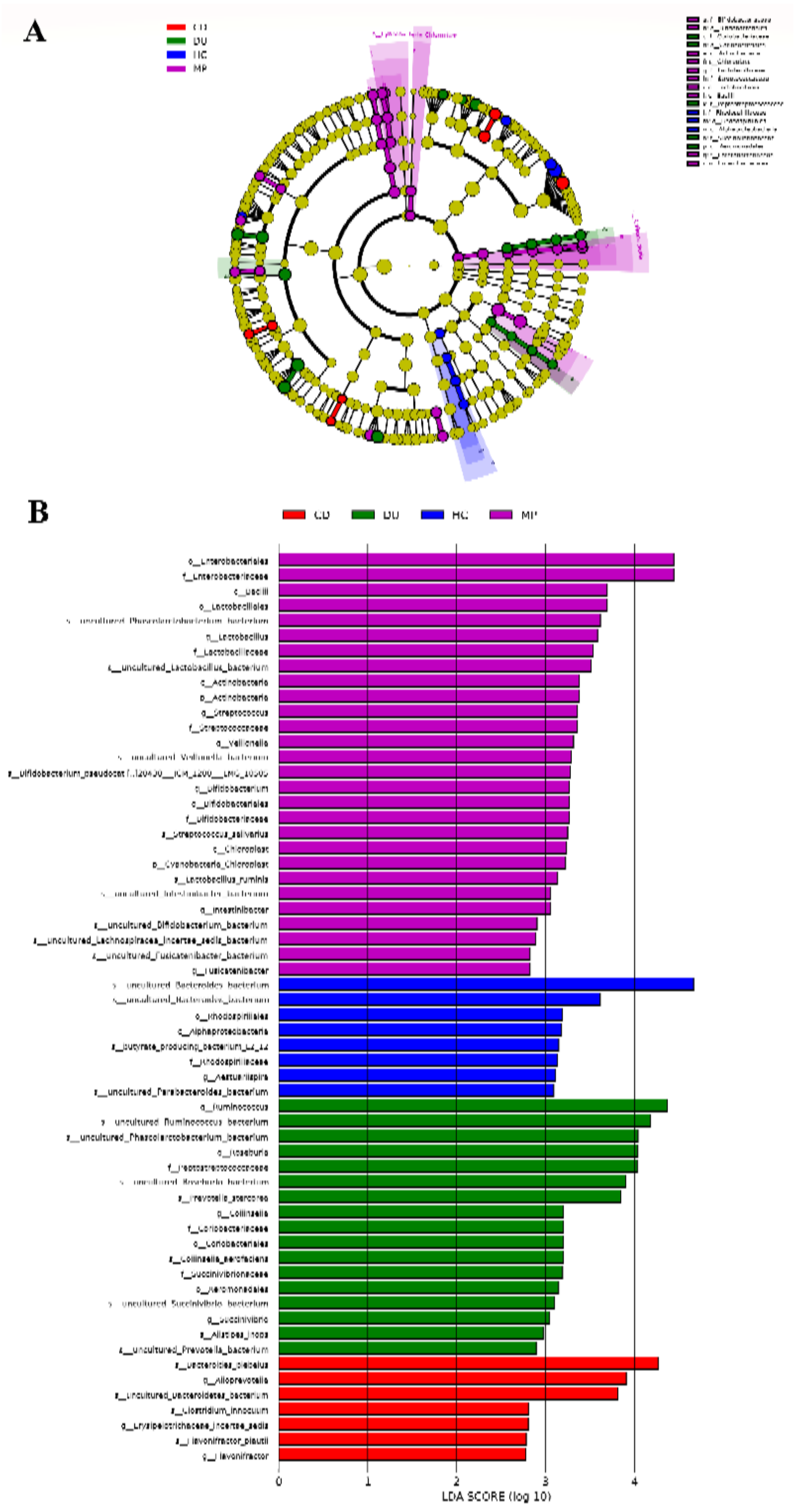

Figure S4. Linear discriminant analysis (LDA) effect size (LEfSe) analysis of signature taxa for DU, CD, MP, and HC. (A) LEfSe evolutionary branching diagram. (B) Bar graph of LDA with an LDA score > 2.0 and *p* < 0.05 among the four groups. The prefixes represent the abbreviations for the taxonomic rank of each taxon: phylum (p), class (c), order (o), family (f), genus (g), and species (s). (CD = compulsory detention participants; MP = MMT patients; DU = drug users; HC = healthy controls).

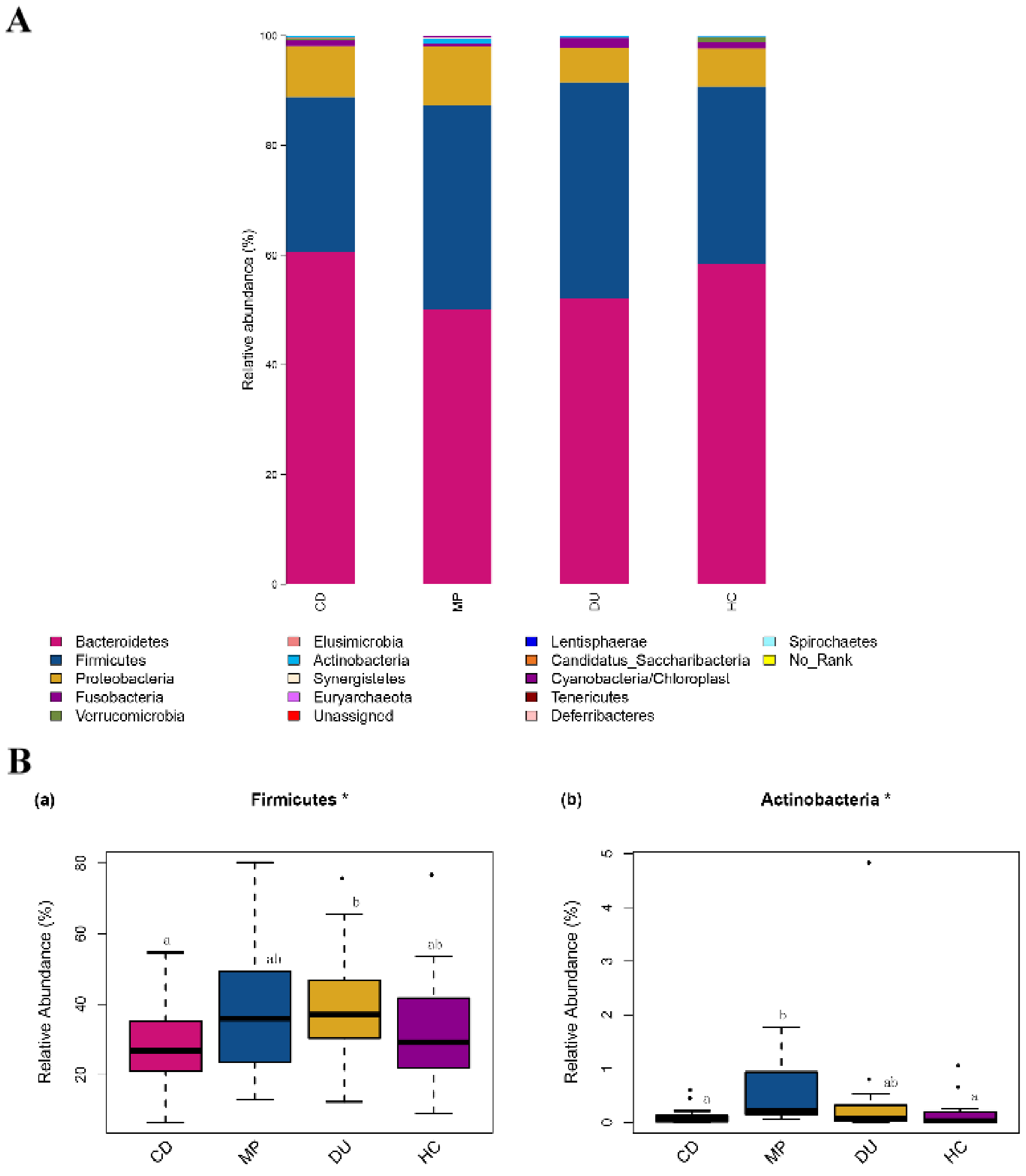

Figure S5. Analysis of community composition and ANOVA comparing differences in the abundance of OTUs at the phylum level among DU, CD, MP, and HC. (A) Relative abundance of bacterial phyla among different groups.. (B) Box plot comparison of taxa with the most significant differences. (* indicates statistical significance identified by ANOVA analysis: $p < 0.05$; $^{a\text{-}c}$ superscripts indicate significant differences between mean values by pairwise comparison between groups, $p < 0.05$; CD = compulsory detention participants; MP = MMT patients; DU = drug users; HC = healthy controls).

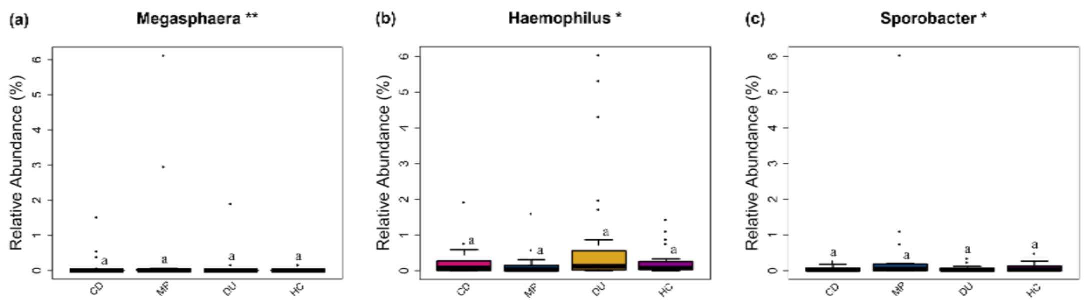

Figure S6. ANOVA comparison of differences in OTUs at the genus level among DU, CD, MP, and HC. (* indicates statistical significance identified by ANOVA analysis: ** $p < 0.01$, * $p < 0.05$; [a] same superscript indicate no significant differences by pairwise comparison between groups; CD = compulsory detention participants; MP = MMT patients; DU = drug users; HC = healthy controls);

Table S1. General demographic information for DU, CD, MP, and HC subjects (n (%)).

Demographic characteristics	CD (n = 28)	MP (n = 16)	DU (n = 27)	HC (n = 28)	χ^2 value	*p*-value
**Gender**					15.469	0.001 *
male	28 (100)	12 (75)	27 (100)	21 (75)		
female	0 (0)	4 (25)	0 (0)	7 (25)		
**Age**					33.957	<0.001 *
≤ 29	13 (46.4)	1 (6.2)	14 (51.9)	13 (46.4)		
30–39	9 (32.1)	3 (18.8)	10 (37.0)	2 (7.1)		
40–49	3 (10.7)	10 (62.5)	3 (11.1)	6 (21.4)		
≥ 50	3 (10.7)	2 (12.5)	0 (0.0)	7 (25.0)		
range (year)	19–54	28–50	18–48	17–59		
**BMI**					5.571	0.782
thin	2 (7.1)	0 (0.0)	3 (11.1)	2 (7.1)		
normal	11 (39.3)	9 (56.2)	15 (55.6)	13 (46.4)		
overweight	12 (42.9)	5 (31.2)	5 (18.5)	9 (32.1)		
obese	3 (10.7)	2 (12.5)	4 (14.8)	4 (14.3)		
**Education level (year)**					32.802	<0.001 *
0–6	8 (28.6)	3 (18.8)	12 (44.4)	0 (0.0)		
7–9	8 (28.6)	8 (50.0)	9 (33.3)	3 (10.7)		
> 9	12 (42.9)	5 (31.2)	6 (22.2)	25 (89.3)		
**Marital status**					7.785	0.254
unmarried	17 (60.7)	5 (31.2)	14 (51.9)	13 (46.4)		
married/cohabitating	8 (28.6)	9 (56.2)	11 (40.7)	15 (53.6)		
divorced/separated	3 (10.7)	2 (12.5)	2 (7.4)	0 (0.0)		
**Parental status**					1.626	0.653
have children	11 (39.3)	9 (56.2)	13 (48.1)	15 (53.6)		
no children	17 (60.7)	7 (43.8)	14 (51.9)	13 (46.4)		

(BMI = body mass index; Research technicians measured the height and weight of participants, then calculated BMI (kg/m^2) as follows: weight (kg)/Height^2 (m). * indicates statistical significance. CD = compulsory detention participants; MP = MMT patients; DU = drug users; HC = healthy controls)

Table S2. Analysis of drug abuse behaviour among CD, MP, and DU.

Drug abuse behaviour	CD (n = 28)	MP (n = 16)	DU (n = 27)	χ^2 value	*p*-value
**Duration of drug abuse (months)**				—	<0.001 *
Med (Q_1, Q_3) ($\alpha = 0.05$)	110 (64, 125)	217 (69, 278)	42 (30, 89)		
Range	31–365	15–373	5–222		
**Drug type, n (%)**				15.340	0.004 *
narcotic	13 (46.4)	16 (100.0)	12 (44.4)		
psychotropic	11 (39.3)	0 (0.0)	10 (37.0)		
narcotic and psychotropic	4 (14.3)	0 (0.0)	5 (18.5)		
**Drug abuse manner, n (%)**				3.257	0.516
injection	3 (10.7)	0 (0.0)	2 (7.4)		
snorting	24 (85.7)	15 (93.8)	25 (92.6)		
Snorting then turning to injection	1 (3.6)	1 (6.2)	0 (0.0)		
**Frequency of forced cessation, n (%)**				41.892	<0.001 *
0 times	0 (0.0)	9 (56.2)	0 (0.0)		
once	22 (78.6)	3 (18.8)	15 (55.6)		
twice	4 (14.3)	2 (12.5)	10 (37.0)		
more than twice	2 (7.1)	2 (12.5)	2 (7.4)		

(* indicates statistical significance. CD = compulsory detention participants; MP = MMT patients; DU = drug users)
